# Preoperative Parathyroid Needle Localization: A Minimally Invasive Novel Technique in Reoperative Settings

**DOI:** 10.1155/2011/487076

**Published:** 2011-08-13

**Authors:** Ryan Winters, Paul Friedlander, Salem Noureldine, Ibrahim Ekaidi, Krzysztof Moroz, Emad Kandil

**Affiliations:** ^1^Department of Otolaryngology, Tulane University School of Medicine, New Orleans, LA 70112, USA; ^2^Department of Surgery, Tulane University School of Medicine, New Orleans, LA 70112, USA; ^3^Department of Pathology, Tulane University School of Medicine, New Orleans, LA 70112, USA

## Abstract

*Background*. Reoperative parathyroid surgery for primary hyperparathyroidism can be challenging. Numerous preoperative localization techniques have been employed to facilitate a more focused surgical exploration. This paper describes a novel, minimally invasive, and highly successful method of parathyroid localization. *Methods*. Patients with recurrent or persistent primary hyperparathyroidism underwent parathyroidectomy following CT scan or ultrasound-guided wire localization of the parathyroid. Accurate placement was confirmed by fine-needle aspiration with immunocytochemistry or PTH washout. The guide wire was left *in situ* to guide surgical excision of the gland. Curative resection was established by monitoring intact serum PTH levels after excision of the adenoma. *Results*. All ten patients underwent successful redo-targeted parathyroidectomy. Nine of the ten patients were discharged on the day of surgery. One patient was observed overnight due to transient postoperative hypocalcemia, which resolved with calcium supplementation. *Conclusion*. Placement of a localization wire via preoperative high-resolution ultrasound or CT can expedite reoperative parathyroid surgery. It allows identification of parathyroid adenoma via a minimally invasive approach, especially in cases where a sestamibi scan is inconclusive.

## 1. Introduction

Reoperative parathyroid surgery is a challenging problem for surgeons. The dense scar tissue and the small size of target lesions necessitate exact surgical localization. Many different techniques have been employed to achieve such precision, including preoperative ultrasound, CT, MRI, and sestamibi scanning. Intraoperative selective venous sampling and serum parathyroid hormone (PTH) monitoring have also been utilized. Sestamibi and ultrasound are commonly used methods, with sensitivities ranging from 53–98% and 56–87%, respectively [1–5]. When sestamibi and ultrasound are used, sensitivity increases to 67–98% [1, 4, 5]. 

While the traditional approach has been bilateral neck exploration with identification of all parathyroid tissue [6], the recent literature has described numerous benefits to focused parathyroidectomy for patients with primary hyperparathyroidism. These included less postoperative pain with decreased need for analgesia, a lower incidence of postoperative hypocalcemia, and better cosmesis [7, 8]. Shorter operative times and lower cost with equivalent results [9] make focused parathyroidectomy extremely attractive. The paper describes a novel, reproducible, and highly successful method of preoperative localization suitable for focused parathyroidectomy.

## 2. Methods

After obtaining approval from the Institutional Review Board of Tulane University, a retrospective review of the charts of 10 nonconsecutive patients over a period of two years, presenting for reoperative treatment of persistent hyperparathyroidism was undertaken.

## 3. Case Series

Of the 10 patients, four were females and six males, with an average age of 50 years old (range: 25–73 years old). All patients had a history of prior neck exploration for primary hyperparathyroidism, with persistent or recurrent hyperparathyroidism after the initial procedure (4 patients with recurrent and 6 with persistent hyperparathyroidism).

**Figure 1:**
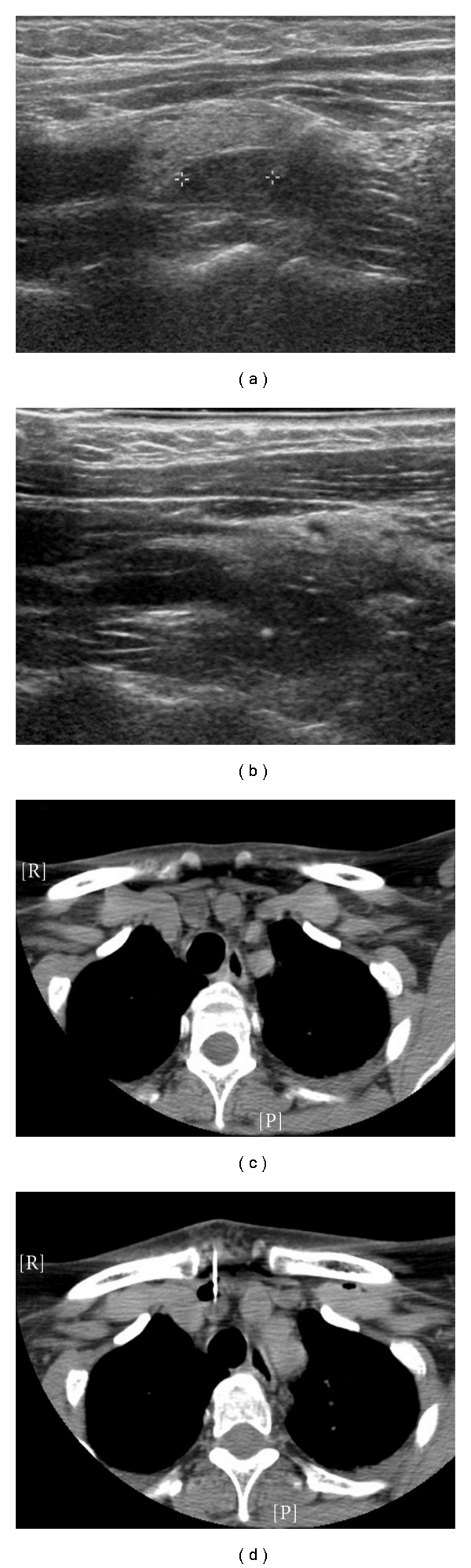
(a) Ultrasound of parathyroid adenoma. (b) Same patient, with guide wire in place (white dot). (c) CT of parathyroid adenoma in retrosternal space. (d) Same patient, with guide wire in place (white line).

After obtaining informed consent from 10 patients, sonography of the neck was performed to identify the parathyroid adenoma. These were compared with other imaging studies, including CT scan, when available. The skin was prepped in the standard fashion and local anesthesia administered. A Homer mammography needle wire device was introduced under ultrasound or CT guidance and the needle tip was guided to the appropriate position within the suspect parathyroid gland (Figure 1). A 22-gauge Chiba needle was passed in a tandem fashion and aspiration of the lesion was performed. The specimens obtained were sent for cytology and/or PTH analysis (Table 1). Subsequently, 0.5 mL methylene blue was instilled through the Homer needle and a hook wire passed through the Homer needle. Placement of the wire tip within the lesion was confirmed by ultrasound. At this point both the Homer needle and wire were left in place and secured, much like in mammographic lesion localization. The patients were then taken directly to the operating room.

**Figure 2:**
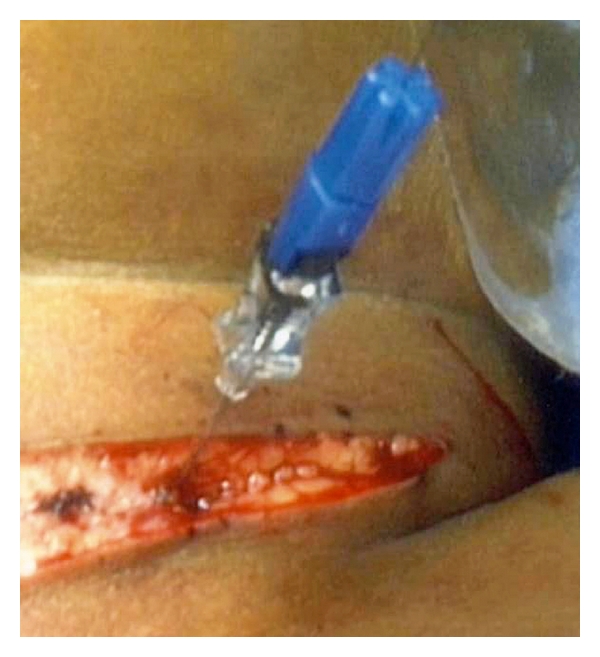
Guide wire *in situ* in operating room. Skin incision has been made to incorporate point of entry of guide wire.

The skin incision was made to include the point of entry of the guide wire (Figure 2), and the wire was followed with meticulous dissection until the lesion was identified both by palpation and the presence of methylene blue. The mass containing the hook wire was subsequently dissected and excised. Intraoperative nerve monitoring was performed in all the patients.

 All patients were successfully treated, with identification and excision of the lesion identified by the guide wire, and despite the vascular nature of parathyroid adenomas, no significant hematomas occurred. In four patients, extremely small hematomas were noted within the parathyroid adenoma on final histology; these did not affect the dissection in any way. Serum PTH levels decreased by at least 50% postoperatively. Curative resection was established in all ten patients by intraoperative monitoring of serum intact PTH levels. Histopathology confirmed the diagnosis of parathyroid adenoma in all 10 patients. The calcium and PTH levels are detailed in Table 1. Seven of the 10 patients had been hyperparathyroid for approximately one year prior to reoperative surgery, with a mean preoperative PTH level of 213.9 pg/mL.

The mean levels fell to 27.84 pg/mL (*s*_*M*_ = 11.2) postoperatively. Nine of the ten patients were discharged home on the day of surgery. One patient was observed overnight because of asymptomatic postoperative hypocalcemia, which was treated with calcium supplementation, and resolved prior to follow-up examination in clinic.

## 4. Discussion

The classic treatment approach for primary hyperparathyroidism has been bilateral neck exploration with identification of all parathyroid glands. Numerous recent reports have shown benefits of more selective approaches, including better cosmesis and decreased risk of nerve injury. These make focused parathyroidectomy techniques very desirable [7, 8]. 

Both ultrasound and CT-guided FNA are well-described, successful techniques for the definitive diagnosis of lesions in the neck. Both techniques can provide a diagnosis in >90% of patients [10, 11]. Surgeon-performed ultrasound has been shown in some studies to increase the rate of localization of parathyroid adenomas, even in the setting of a nonlocalizing sestamibi scan [12]. Part of this increase in success may be due to the real-time nature of surgeon-performed ultrasound, allowing a more immediate and thorough sonographic examination of the area of interest at the time of surgery. 

Benefits from real-time examination are also apparent using the wire localization technique described in this study. The high degree of accuracy afforded by ultrasonographic examination immediately prior to surgery allows placement of the needle and guide wire, with confidence.

Only six patients out of nine were confirmed to have parathyroid tissue on cytopathological examination at time of biopsy. In the other four patients, cytology was nondiagnostic. All patients had PTH washout, which confirmed the correct localization of the parathyroid tissue. Frasoldati and colleagues [13] showed that FNA-PTH washout more than 101 pg/mL had a 100% sensitivity and specificity for verification of parathyroid tissue. One patient from our current series underwent this procedure during her pregnancy and we reported recently a successful outcome in this patient [14].

 A review of the literature revealed a total of four additional cases where wire or needle localization was utilized for surgery in the neck, however all of them utilized CT guidance [15–17]. The overall technique is similar, using image guidance to precisely place a guide wire into the target lesion. The ability to follow the wire intraoperatively avoids unnecessary trauma to other structures from dissection and alleviates the need for a more extensive operation.

This guide-wire technique is an effective method to prevent damage to the recurrent laryngeal nerve (RLN). This is of utmost importance in reoperative cases, where RLN injury rates as high as 10% have been reported [18]. The technical difficulties posed by the scar tissue and distorted anatomy in the reoperative neck are such that even traditional intraoperative nerve monitoring has not always decreased the rate of RLN injury in these patients [19]. Allowing the surgeon to follow the path of an image-guided wire to the target lesion, combined with standard nerve monitoring, has facilitated avoiding recurrent laryngeal nerve injury. 

Preoperative image-guided Homer needle wire placement and methylene blue injection for reoperative hyperparathyroid patients was able to correctly identify all lesions in our series. While preoperative wire placement is certainly not indicated in every reoperative patient, this technique provides another tool in the armamentarium available to physicians treating hyperparathyroidism in high-risk patients with prior neck surgery.

## Figures and Tables

**Table 1:** Results of ultrasound-guided FNA, PTH washout before guide wire placement and pre/postoperative calcium and PTH levels after parathyroid adenoma removal using guide wire localization.

Patient	Preop Ca (mg/dL)	Postop Ca (mg/dL)	Preop PTH (pg/mL)	Postop PTH (pg/mL)	Fna ± for parathyroid	PTH washout
Patient 1:	10.1	8.9	103	4	−ve	Unavailable
Patient 2:	11.4	8.7	148	31	+ve	103
Patient 3:	11.1	8.8	158	80	+ve	Unavailable
Patient 4:	11.7	8.3	284	15.4	+ve	180,000
Patient 5:	11.2	8.5	212	8	+ve	>2500
Patient 6:	10.4	9.1	121	27	−ve	204,727
Patient 7:	10.7	8.9	149	32	−ve	1816
Patient 8:	10.4	8.8	613	<5	+ve	952
Patient 9:	8.9	8.6	95	29	−ve	<60
Patient 10:	10.5	9.0	107	47	+ve	3433
